# DUSP3 restrains the progression and stemness property of osteosarcoma through regulating EGFR/STAT3/SOX2 axis

**DOI:** 10.7150/ijbs.101137

**Published:** 2025-01-01

**Authors:** Zhun Wei, Di Zheng, Kezhou Xia, Xinghan Huang, Wenda Liu, Zicheng Wei, Weichun Guo

**Affiliations:** Department of Orthopedics, Renmin Hospital of Wuhan University, Hubei Province, Wuhan, 430060, China.

**Keywords:** DUSP3, osteosarcoma, stemness, proliferation, therapeutic

## Abstract

**Background:** Dual-specificity phosphatase 3 (DUSP3) is a small-molecule dual-specificity phosphatase whose function has not yet been elucidated. This study investigated the effects of DUSP3 on the biological behavior of osteosarcoma and its potential mechanisms.

**Methods:** We performed bioinformatics analysis of DUSP3 using “The Cancer Genome Atlas” and “The Tumor Immune Estimation Resource” databases. The impact of DUSP3 on osteosarcoma biological behavior was evaluated using CCK-8, wound-healing, transwell invasion, and tumor sphere formation assays. Immunoprecipitation assays confirmed the interaction between two proteins. We then established a nude mouse transplantation tumor model to examine the in *vivo* effects of DUSP3 on osteosarcoma.

**Results:** Overexpression of DUSP3 significantly inhibited osteosarcoma cell proliferation, migration, invasion, and stemness. Conversely, DUSP3 knockdown yielded the opposite results. In an animal model, we administered subcutaneous injections of 143B osteosarcoma cells overexpressing DUSP3, and the results indicated that the overexpression of DUSP3 impaired osteosarcoma growth.

**Conclusion:** Our findings indicate that DUSP3 could be an independent prognostic determinant in individuals diagnosed with osteosarcoma. Through modulating the EGFR/STAT3/SOX2 axis, DUSP3 restrains osteosarcoma cell growth, migration, invasion, and stemness. Therefore, targeting DUSP3 may serve as an effective therapeutic target in osteosarcoma treatment.

## Introduction

Osteosarcoma, originating from bone-forming mesenchymal cells, is the most prevalent malignant bone tumor in children 1. Its early stage often presents with localized swelling and pain, making detection challenging. In advanced stages, it renders bones prone to fractures and is often associated with pulmonary metastasis 2. Neoadjuvant chemotherapy, comprising preoperative chemotherapy, total surgical excision of the lesion, and postoperative chemotherapy, is the main course of treatment. 3. Despite current advanced treatments increasing the 5-year survival rate for individuals with low-grade osteosarcoma to 70%, treatment outcomes for high-grade osteosarcoma remain suboptimal 4, 5, highlighting the urgent need to explore new therapeutic tools and identify better therapeutic targets for osteosarcoma treatment.

Cancer stem cells (CSCs) are tumor cells capable of self-renewal, promoting tumorigenesis, driving metastasis, and enhancing resistance to cancer treatments, leading to poor patient prognosis 6, 7. Current research has shown the critical role of tumor cell stemness in the pathogenesis of various cancers, including colorectal cancer, hepatocellular carcinoma, lung cancer, and skin cancer 8-11. Chi et al. identified that AGR3 promotes colorectal cancer progression by regulating the Wnt/β-catenin signaling pathway, enhancing β-catenin nuclear translocation, and upregulating stemness-related genes 12. CCAT2 controls the aggressiveness of triple-negative breast CSCs by promoting OCT4-PG1 expression and upregulating the Notch signaling pathway 13. There has also been a growing interest in the function of stemness in osteosarcoma. Chen et al. suggested that MAFB is overexpressed in osteosarcoma and maintains tumor self-renewal potential and malignancy by upregulating the stem cell regulator SOX9 at the transcriptional level 14. The WAC-AS1 protein binds to miR-5047 and increases SOX2 expression to promote osteosarcoma cell proliferation and stemness 15. CircPIP5K1A promotes osteosarcoma stemness through the miR-515-5p/YAP axis, indicating its viability as a therapeutic strategy for osteosarcoma 16. Pimozide suppresses osteosarcoma cell proliferation and stemness via phosphorylating STAT3 and STAT5, according to a pharmacological investigation 17. At the same time, studies have shown that conventional chemotherapy drugs can induce the transformation of osteosarcoma cells to a stem cell-like phenotype. This is related to activation of Wnt/β-catenin signaling, upregulation of pluripotency factors and detoxification systems, ultimately leading to chemotherapy failure 18. Therefore, a better understanding of cancer cell stemness is critical for developing effective clinical treatments for osteosarcoma.

Dual-specificity phosphatase 3 (DUSP3), also known as Vaccinia H1-related phosphatase, is a small-molecule dual-specificity phosphatase whose function remains unclear in osteosarcoma 19. In this work, the underlying molecular mechanisms of DUSP3's effects on osteosarcoma biological behavior were examined.

This present study found a decreased DUSP3 expression in human osteosarcoma tissues, and DUSP3 may function as an independent prognostic factor in osteosarcoma patients. To validate these results, we constructed two stable transgenic cell lines. Functional assays showed that the knockdown or upregulation of DUSP3 in osteosarcoma cells significantly affected their proliferation, migration, invasion, and stemness. We found that DUSP3 affected osteosarcoma proliferation, migration, and invasion by regulating the Epidermal Growth Factor Receptor (EGFR)/STAT3/SOX2 axis. Overall, our study revealed a new mechanism of stemness regulation in osteosarcoma. Targeting the EGFR/STAT3/SOX2 axis may be a promising therapeutic approach for treating osteosarcoma.

## Materials and Methods

### Sample acquisition

The acquisition of human specimens was authorized by the ethics committee of Renmin Hospital of Wuhan University. Osteosarcoma tissues in human were acquired from Renmin Hospital of Wuhan University between March 2020 and May 2024 (**Table 1**). After surgical resection, all human samples were soaked in liquid nitrogen or 4% paraformaldehyde for subsequent experiments.

### Bioinformatic analysis

Using DUSP3 as keywords, the expression levels of DUSP3 in a variety of malignancies were demonstrated in Tumor Immune Estimation Resource (TIMER) database (https://cistrome.shinyapps.io/timer/). With information from the Cancer Genome Atlas (TCGA) database, the receiver operating characteristic (ROC) curves, Cox regression analyses, and Kaplan-Meier survival analysis were carried out. Molecular docking was performed using Pymol 2.5.4 and AutoDock Tools 1.5.7. Using DUSP3 and EGFR as keywords, the protein structures were retrieved from the UniProt database, the human species were screened, and the AlphaFold-predicted structures of DUSP3 and EGFR were selected as receptor and ligand proteins for protein-protein docking. The binding interface of protein-protein complexes was comprehensively described and systematically analyzed, with details of the interaction supplemented using Pymol. Hydrophobic interactions, hydrogen bonds, and salt bridges between the two proteins were demonstrated.

### Cell culture and transfection

Procell Technology (Wuhan, China) supplied the U2OS, MG63, and HOS cells, while hFOB1.19 and 143B cells were obtained from Servicebio Technology (Wuhan, China). 10% or 20% fetal bovine serum (FBS) (Gibco, USA) and 1% antibiotics (100 units/mL of streptomycin and penicillin) were added to the medium and were used to cultivate osteosarcoma cells. The cells were then incubated at 37 °C with 5% CO_2_. The shRNAs used for transfection were constructed by OBio Technology (Shanghai, China): shEGFR: AGAATGTGGAATACCTAAGG; shDUSP3: CGTGAGGCAGAACCGTGAGAT; shNC: TTCTCCGAACGTGTCACGT. For lentiviral transfection, the CDS regions of DUSP3 and EGFR were cloned into a lentiviral vector as previously described 20. Recombinant lentivirus overexpressing DUSP3 and silencing DUSP3 were constructed by OBio Technology (Shanghai, China). After transfection, stable transfected cells were screened out using 5 μg/mL puromycin.

### Cell counting kit-8 (CCK-8) assay

Cells were incubated for 24, 48, and 72 hours after being uniformly seeded in 96-well plates. At each time point, CCK-8 reagent (Servicebio, Wuhan, China) was added to each well and incubated for 1 h. Subsequently, an enzyme marker was then used to detect the OD450 value of cells in each well.

### 5-Ethynyl-2'-deoxyuridine (EdU) staining assay

A EdU detection kit (Servicebio, Wuhan, China) was utilized following the manufacturer's instructions. First, cells in the logarithmic growth phase were taken and inoculated in a 6-well plate. EdU medium was then prepared and added to the cells for incubation for 2 hours. Then the cells were washed with PBS, fixed with 4% paraformaldehyde, and neutralized with glycine. Finally, cells were stained with the EdU kit and DAPI. EdU-positive cells were observed under an inverted microscope (Olympus, Tokyo, Japan).

### Wound healing assay and transwell invasion assay

First, a marker pen was used to draw uniform horizontal lines on the back of the 6-well plate, then 5×10^5^ cells was added to the wells. When the cells are evenly attached, a 1 ml pipette tip was utilized to scratch the cell layer along the line on the back of the plate. After the scratching is completed, wash the cells 3 times with sterile PBS, then replace with serum-free culture medium, and place the cells in a 37℃, 5% CO_2_ incubator for culture. We then measured and examined the scratches' diameters. For the transwell invasion assay, transwell chambers (Corning, USA) and Matrigel (Corning, USA) were utilized. Culture medium with 20% FBS was added to the bottom chamber, while Matrigel was added to the top chamber. Subsequently, 200 μL of 1×10^5^ cell suspension was added to the upper chamber and incubated at 37 °C with 5% CO_2_. The upper chamber was removed, fixed with 4% paraformaldehyde, and stained with crystal violet.

### Tumor sphere formation

Cells (4×10^4^) were resuspended in DMEM/F12 medium containing β-FGF (10 ng/mL), B27, and EGF (20 ng/mL each) (all from Proteintech, Wuhan, China), which were spread uniformly on ultra-low adhesion six-well plates. The cells were then cultivated for 10 days at 37 °C and 5% CO_2_. The tumorspheres were imaged using an inverted microscope (Olympus, Tokyo, Japan).

### Western blot analysis

Cells or tissues were lysed using RIPA buffer (Servicebio, Wuhan, China) to extract total protein. A BCA kit (EpiZyme, Shanghai, China) was then used to assess the protein concentration. The proteins underwent electrophoresis separation and then electrotransfer onto a PVDF membrane. The membrane was blocked and cleaned with TBST before being incubated with the matching primary antibody (**Table 2**) for the entire night and the secondary antibody for one hour the following day. The bands were visualized using an EpiZyme (Shanghai, China) ECL equipment.

### Immunoprecipitation (IP)

Initially, 293T and 143B transfected cells were gathered. ProteinA/G magnetic beads (EpiZyme, Shanghai, China) conjugated with antibodies were added to the IP lysate after treatment, the mixture was then incubated for an additional night at 4 °C. The proteins were heated to 100 °C in a metal bath after the magnetic beads were collected the following day and cleaned with IP buffer. Protein expression was detected using western blotting.

### Immunohistochemical (IHC) staining assays

First, the tissues were fixed in 4% paraformaldehyde for 3-4 hours. Then, dehydrate it in different concentrations of alcohol and anhydrous ethanol. Embed the tissue in paraffin and slice it, the thickness is generally 4-5 microns. The DAB kit (CST, USA) was applied for detection after antigen repair on the slice and incubation with the corresponding primary and secondary antibodies.

### Animal studies

Our animal research was approved by The Ethics Committee of Renmin Hospital of Wuhan University. Nude mice (male) were obtained from Shulaibao Biotech (Wuhan, China) and divided into two groups at random. The two groups were then given subcutaneous injections of 143B osteosarcoma cells that had been stably transfected (LV-Control and LV-DUSP3). Tumor volumes were measured weekly. The animals were sacrificed after 4 weeks under anesthesia with 2% pentobarbital sodium (150 mg/kg). All the tumors were then measured and kept in liquid nitrogen or 4% paraformaldehyde for subsequent experiments. For the lung metastasis assay, two groups of tumor cells were injected into mice via the tail vein. After 2 weeks, mice were intraperitoneally injected with 15 mg/mL luciferase and euthanized 15 min later. The lung tissue was removed, and the distribution and extent of lung metastases were observed using the darkroom imaging platform IVIS Lumina III (PerkinElmer, USA).

### Statistical methods

The means ± standard deviations (SD) of all the data are presented. The software GraphPad Prism (version 8.0) was applied to conduct quantitative statistical analysis. Every experiment was carried out three times. P < 0.05 was used to indicate statistical significance.

## Result

### DUSP3 was downregulated in osteosarcoma and its lower expression predicts poor prognosis in osteosarcoma

**Figure 1:**
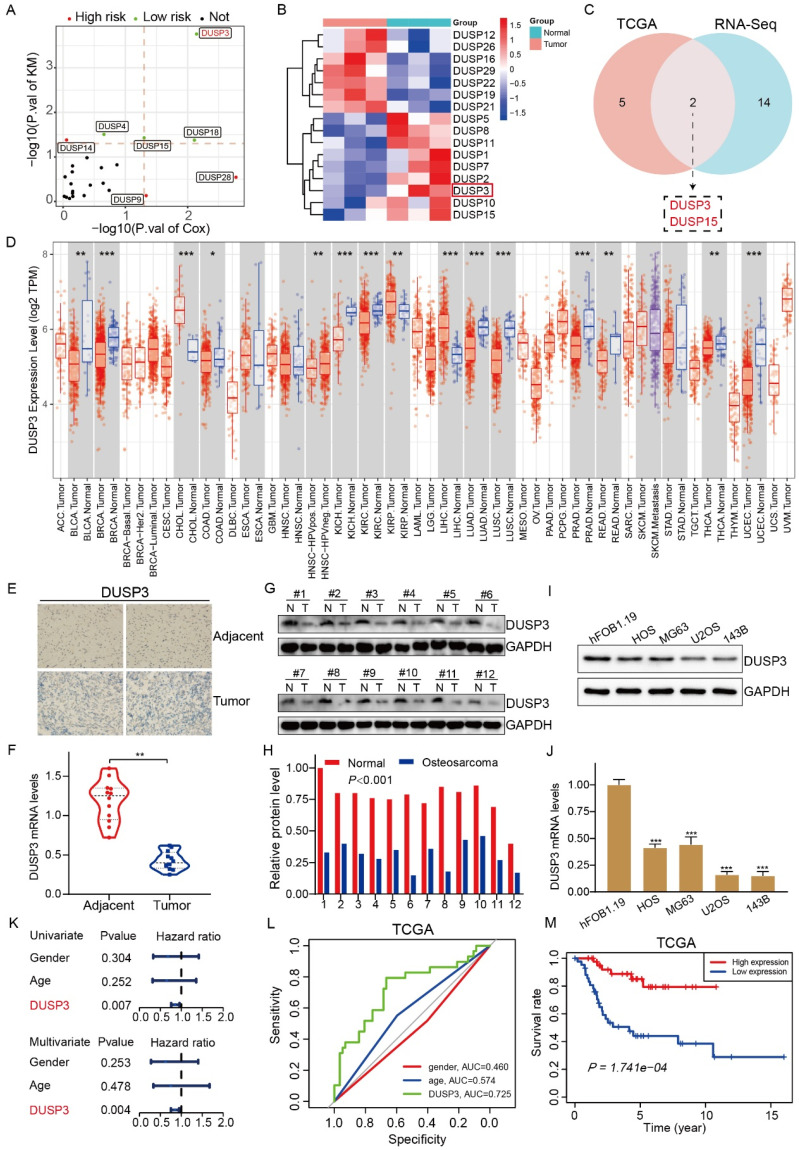
DUSP3 is downregulated in osteosarcoma and its lower expression indicates poorer prognosis in osteosarcoma. (A) The volcano diagram displays the genes from the DUSP family linked to osteosarcoma patients' prognosis. (B) Heatmap showing differentially expressed DUSP family genes in osteosarcoma and adjacent tissues. (C) The Venn diagram shows the DUSP family genes associated with patient prognosis in the TCGA database and transcriptome sequencing. (D) Expression levels of DUSP3 in different tumors in the TIMER database. Abbreviations: https://gdc.cancer.gov/resources-tcga-users/tcga-code-tables/tcga-study-abbreviations. (E-F) DUSP3 expression level and in osteosarcoma and paracancerous tissues were determined using immunohistochemistry (IHC) and qRT-PCR. (G-H) The level of DUSP3 expression in human samples was determined using the Western blot assay. (I-J) Protein and mRNA levels of DUSP3 in hFOB1.19, HOS, MG63, U2OS, and 143B cells. (K) Cox regression analyses of DUSP3 in TCGA database. (L) The precision with which DUSP3 predicted the prognosis of a patient was assessed using ROC curve analysis. (M) Kaplan-Meier survival analysis of DUSP3 based on TCGA database. For analysis of group differences, student's t-test (two groups) and one-way ANOVA (more than two groups) were used. All data are demonstrated as means ± standard deviations (SD). *P <0.05, **P <0.01, ***P <0.001.

Through analysis of the clinical information in TCGA database, we obtained DUSP family genes related to patients' survival (**Figure 1A**). We performed transcriptome sequencing of the collected samples and found that several DUSP-related genes were differentially expressed (**Figure 1B**). By combining the obtained data, we found that DUSP3 and DUSP15 were differentially expressed in osteosarcoma and associated with patients' prognosis (**Figure 1C**). By searching TIMER database, we discovered that the expression of DUSP3 varied throughout various tumors (**Figure 1D**). However, the expression level of DUSP3 in osteosarcoma remains unclear. To further investigate this, immunohistochemistry and qRT-PCR was applied to detect the levels of DUSP3 expression in the tumor and adjacent tissues (**Figure 1E-F**). The protein levels of DUSP3 in twelve pairs of human osteosarcoma and paraneoplastic tissue samples were measured using western blot analysis. (**Figure 1G-H**). It was observed that the osteosarcoma tissues had a decreased level of DUSP3 protein expression in comparison to the adjacent tissues. Additionally, we detected DUSP3 expression in osteosarcoma cell lines. U2OS and 143B had the lowest level of DUSP3 compared with hFOB1.19 normal osteoblast cells (**Figure 1I-J**).

Univariate and multivariate Cox regression analysis demonstrate that, unlike other indices like sex and age, the expression level of DUSP3 is an independent prognostic factor (**Figure 1K**). ROC curves were plotted based on TCGA data, revealing that DUSP3 is a meaningful prognostic marker in osteosarcoma (AUC=0.725) (**Figure 1L**). The Kaplan-Meier survival analysis showed that the group with higher DUSP3 expression had a higher survival rate than the group with lower expression (**Figure 1M**). Overall, these results show that DUSP3 is downregulated in osteosarcoma and that lower expression of the protein is linked to poorer patient outcomes.

### Upregulation of DUSP3 impairs the proliferation, migration and invasion of osteosarcoma cells

**Figure 2:**
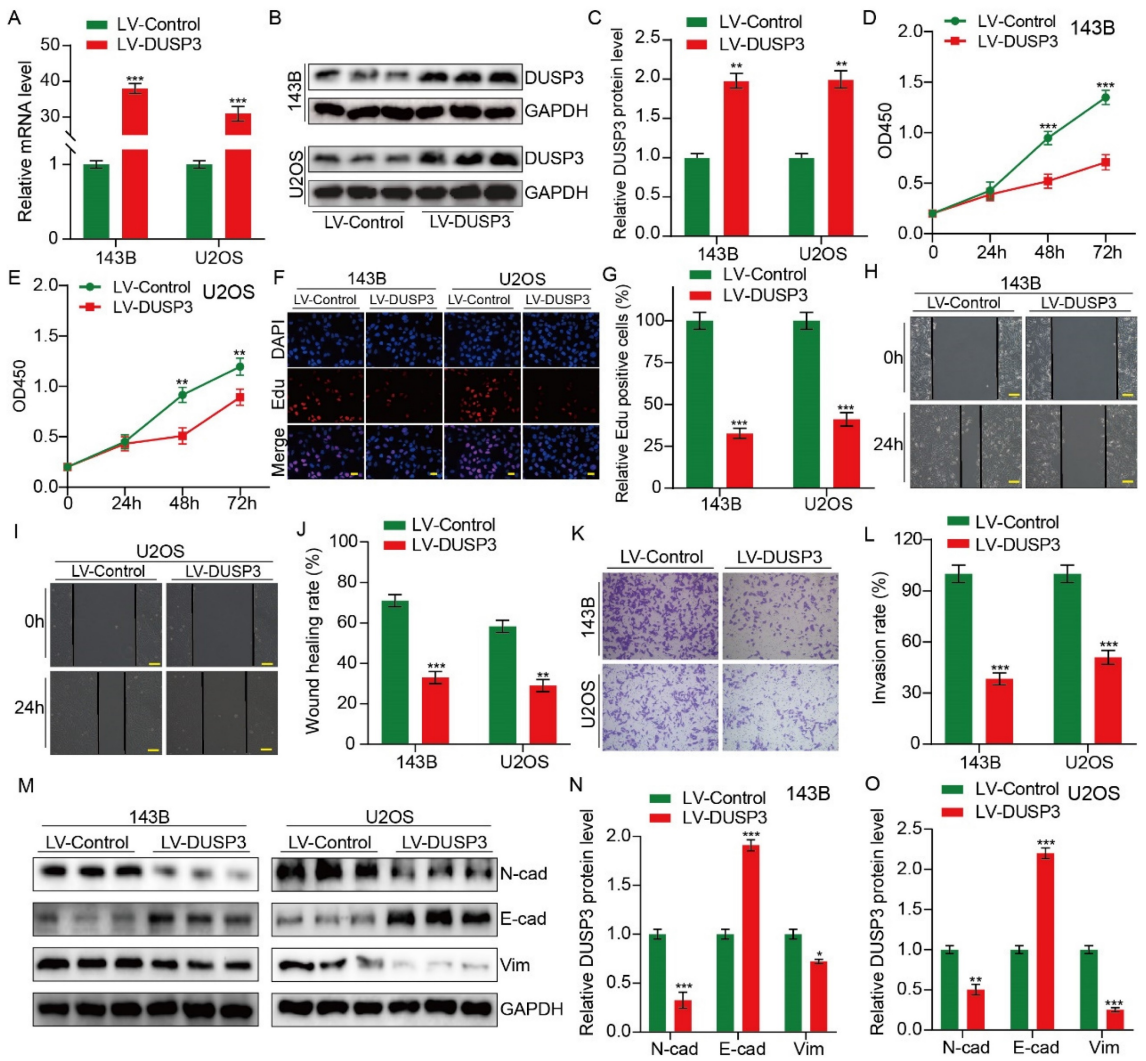
Upregulation of DUSP3 impairs osteosarcoma cell proliferation, migration, and invasion. (A-C) Western blot and qRT-PCR tests were used to confirm that DUSP3 was stably overexpressed. (D-E) The CCK-8 assay was used to compare the proliferative capacity of osteosarcoma cells. (F-G) The number of EdU-positive cells was significantly reduced after overexpression of DUSP3. (H-J) Wound healing assay was applied to measure the migration capabilities of 143B and U2OS cells. (K-L) In transwell assay, overexpression of DUSP3 significantly inhibited the invasion of osteosarcoma cells. (M-O) EMT-related genes, such as N-cadherin, E-cadherin, and Vimentin, were detected by Western blot assay. Student's t-test (two groups) and one-way ANOVA (more than two groups) were employed to identify group differences. The means ± standard deviations (SD) are used to illustrate all data. *P <0.05, **P <0.01, ***P <0.001.

We then hypothesized that DUSP3, which is strongly correlated with the prognosis of osteosarcoma patients, may influence osteosarcoma cells' biological activities. Stably transfected osteosarcoma cells (143B and U2OS) overexpressing DUSP3 were constructed using recombinant lentivirus. qRT-PCR and western blot assay were used to detect the transfection efficiency (**Figure 2A-C**). The CCK-8 assay suggested that DUSP3 upregulation downregulated the OD450 value of osteosarcoma cells (**Figure 2D-E**). In EdU assays, EdU-positive cells significantly decreased in the DUSP3 overexpressing group (**Figure 2F-G**). These findings suggested that osteosarcoma cell proliferation was markedly suppressed by DUSP3 overexpression. After then, the capability of osteosarcoma cells to migrate was assessed using a wound-healing experiment. Our results demonstrated that DUSP3 overexpression attenuated the migratory capability of osteosarcoma (**Figure 2H-J**). DUSP3 overexpression dramatically reduced osteosarcoma cell invasion in the transwell invasion assay, as evidenced by the fact that LV-DUSP3 group cells invaded less than LV-control group cells (**Figure 2K-L**). Additionally, we identified proteins associated with the epithelial-mesenchymal transition (EMT). Our results demonstrated a drop in the expression levels of N-cadherin and Vimentin, whereas there was an upregulation of E-cadherin (**Figure 2M-O**), indicating that osteosarcoma cell invasion, migration, and proliferation are hampered by DUSP3 overexpression.

### DUSP3 silence promoted the proliferation, migration, and invasion of osteosarcoma cells

**Figure 3:**
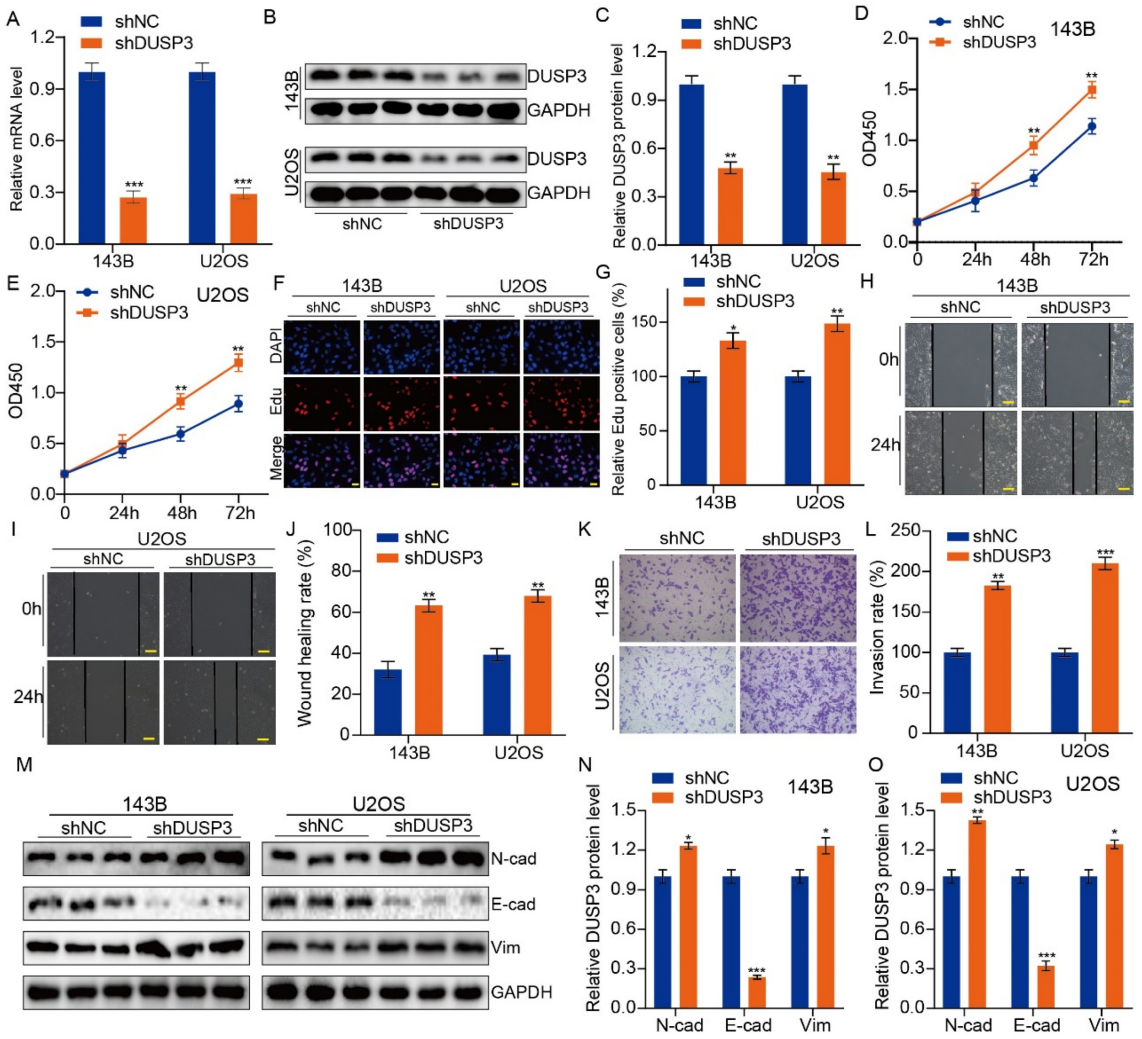
DUSP3 knockdown promotes the progression of osteosarcoma cells. (A-C) qRT-PCR and western blot assays confirmed the efficiency of transfection. (D-E) Reduced expression of DUSP3 increases osteosarcoma cells' ability to proliferate. (F-G) The number of EdU-positive cells was significantly increased after DUSP3 knockdown. (H-J) The capability of osteosarcoma cells to migrate is enhanced by DUSP3 downregulation. (K-L) Silencing of DUSP3 markedly promotes the invasion ability of osteosarcoma cells. (M-O) EMT-related genes, including N-cadherin, E-cadherin, and Vimentin, were detected. For analysis of group differences, student's t-test (two groups) and one-way ANOVA (more than two groups) were utilized. All data are demonstrated as means ± standard deviations (SD). *P <0.05, **P <0.01, ***P <0.001.

Given that overexpression of DUSP3 restrains the progression of osteosarcoma, we hypothesized that silencing DUSP3 might have the opposite effect. U2OS and 143B osteosarcoma cells were transfected with shNC and shDUSP3, respectively. DUSP3 expression levels were measured (**Figure 3A-C**). EdU staining and CCK-8 assays suggested that DUSP3 downregulation significantly promoted osteosarcoma cell proliferation (**Figure 3D-G**). Similarly, following DUSP3 knockdown, osteosarcoma cells' capacity to migrate and invade was noticeably increased in the wound-healing and transwell invasion experiments (**Figure 3H-L**). EMT-related proteins were identified. We observed a significant decrease in E-cadherin expression alongside increased levels of N-cadherin and Vimentin (**Figure 3M-O**). Thus, osteosarcoma cells proliferate, migrate, and invade more readily when DUSP3 is silenced.

### DUSP3 negatively regulates stemness of osteosarcoma cells

**Figure 4:**
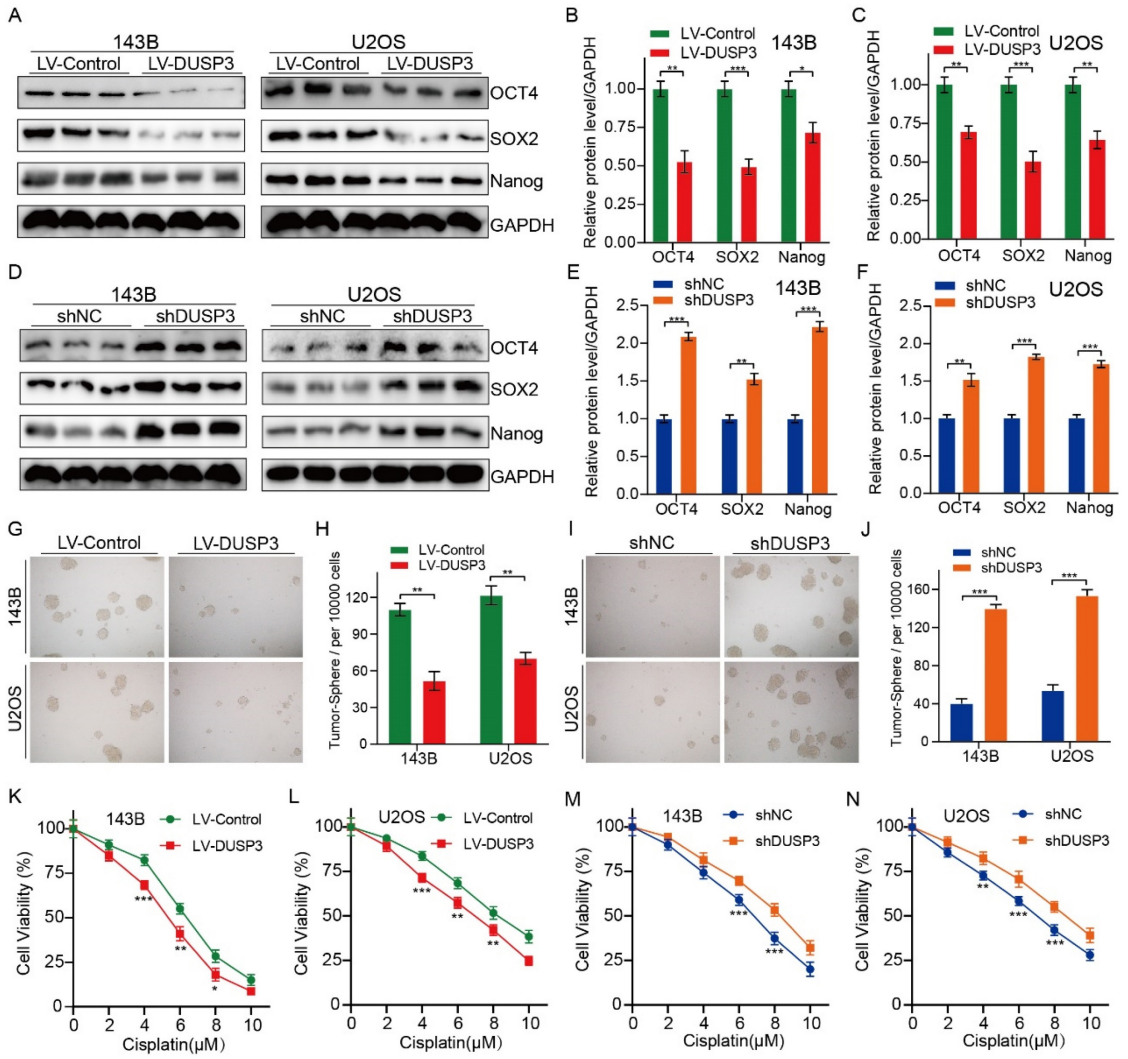
DUSP3 negatively regulates osteosarcoma cell stemness. (A-C) Overexpression of DUSP3 decreased the expression level of cell stemness-related genes, including OCT4, SOX2, and Nanog. (D-F) DUSP3 knockdown markedly increased the protein expression level of OCT4, SOX2, and Nanog. (G-H) Overexpression of DUSP3 significantly impaired the cancer stem cell population of osteosarcoma cells. (I-J) DUSP3 knockdown enriched cancer stem cell population in osteosarcoma cells. (K-N) After treatment of osteosarcoma cells with different concentrations of cisplatin, its viability was detected using CCK-8 assay. Student's t-test (two groups) and one-way ANOVA (more than two groups) were performed to identify group differences. The means ± standard deviations (SD) are used to illustrate all data. *P <0.05, **P <0.01, ***P <0.001.

We then speculated that DUSP3 regulates osteosarcoma cell stemness. We detected the expression levels of the stemness markers including OCT4, SOX2, and Nanog, finding that DUSP3 upregulation significantly decreased their expression levels (**Figure 4A-C**); conversely, DUSP3 downregulation resulted in a noticeably higher levels of cancer stemness markers (**Figure 4D-F**). Tumorsphere formation ability was measured. DUSP3 overexpression significantly decreased the cell population in 143B and U2OS osteosarcoma cells (**Figure 4G-H**); similarly, DUSP3 knockdown enriched the cell population (**Figure 4I-J**). Given that CSCs promote tumorigenesis, metastasis, and chemoresistance 21, 22, we further investigated whether DUSP3-mediated stemness influences the sensitivity of osteosarcoma cells to chemotherapy drugs. The CCK-8 assay indicated that DUSP3-mediated stemness regulated the sensitivity of osteosarcoma cells to cisplatin (**Figure 4K-N**), suggesting that DUSP3 negatively regulates osteosarcoma cells stemness, thus improving their sensitivity to cisplatin.

### DUSP3 attenuates osteosarcoma cells progression via inhibiting STAT3/SOX2 axis

**Figure 5:**
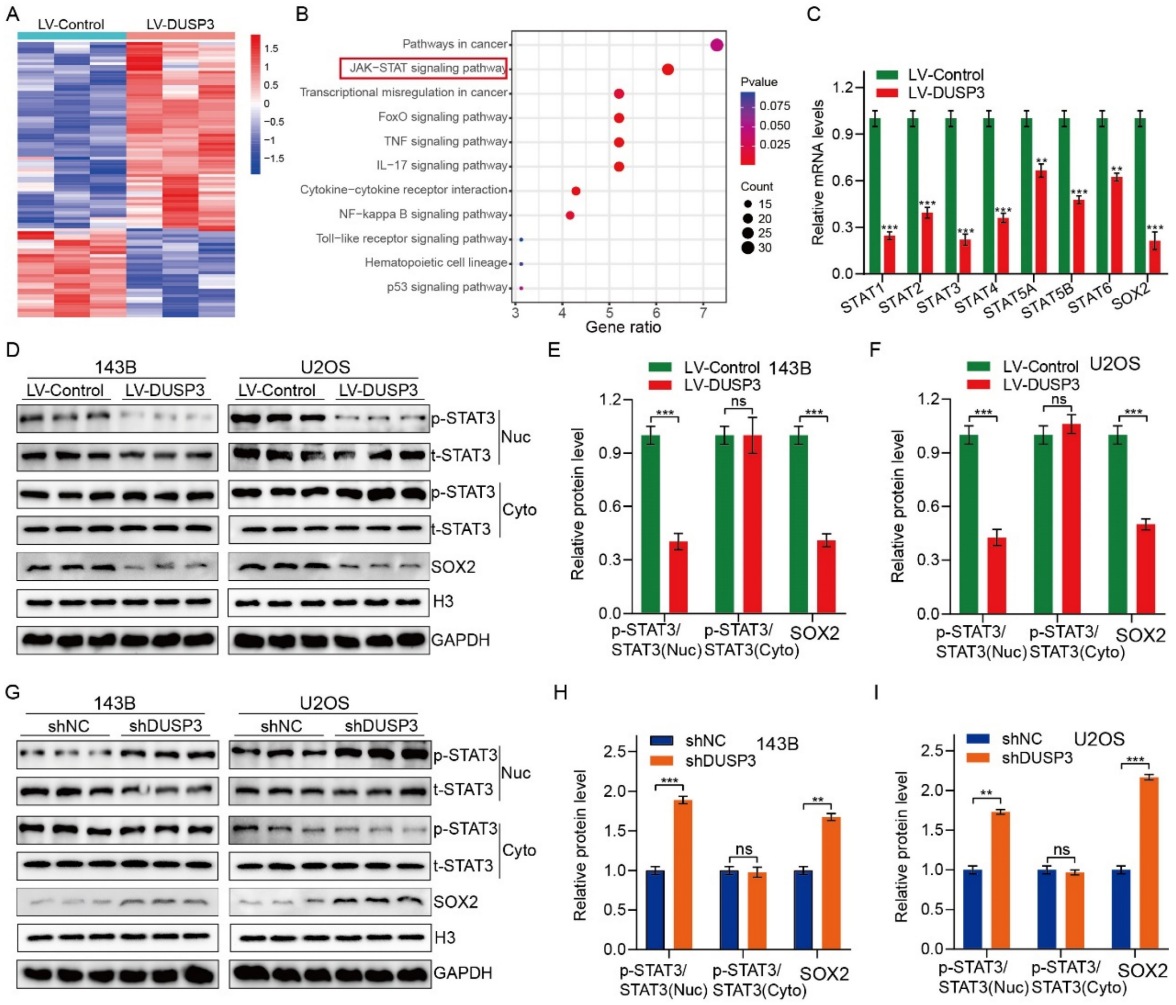
DUSP3 impairs the progression of osteosarcoma cells by inhibiting STAT3/SOX2 axis. (A) A heatmap displaying genes associated with DUSP3. (B) KEGG enrichment analysis shows a strong correlation between DUSP3 expression and the JAK/STAT signaling pathway. (C) qRT-PCR was performed to detect mRNA expression levels of JAK/STAT pathway-related indicators. (D-F) Overexpression of DUSP3 decreased the expression level of p-STAT3 and SOX2. (G-I) DUSP3 knockdown significantly increased p-STAT3 and SOX2 expression levels. For analysis of group differences, student's t-test (two groups) and one-way ANOVA (more than two groups) were used. All data are demonstrated as means ± standard deviations (SD). **P <0.01, ***P <0.001.

We further investigated the specific molecular mechanisms by which DUSP3 affects osteosarcoma cell genesis, development, and stemness. RNA-seq technology was applied to 143B osteosarcoma cells with or without DUSP3 overexpression. We then screened for differential genes between high and low DUSP3 expression samples and performed KEGG (Kyoto Encyclopedia of Genes and Genomes) enrichment analysis, which showed that the JAK/STAT signaling pathway was closely associated with DUSP3 expression levels in osteosarcoma (**Figure 5A-B**). Prior research has indicated that the regulation of tumor cell stemness is significantly influenced by the STAT3/SOX2 signaling axis 23. Meanwhile, STAT3 is a direct upstream molecule of SOX2 and STAT3 regulates the transcriptional level of SOX2 in various tumors 24, 25. We further examined the mRNA levels of relevant molecules and found that the STAT3/SOX2 signaling axis was significantly inhibited in our model (**Figure 5C**). We investigated the relationship between DUSP3 and the STAT3/SOX2 axis by measuring t-STAT3, p-STAT3, and SOX2 expression levels. DUSP3 upregulation significantly decreased the expression of p-STAT3 and SOX2 (**Figure 5D-F**), while its knockdown increased their expression (**Figure 5G-I**). Subsequently, we speculated that whether the effect of DUSP3 was mediated through the STAT3/SOX2 axis. Stattic, an inhibitor of STAT3, was used to treat the osteosarcoma cells. Stattic treatment notably restrained the promotion effect of DUSP3 knockdown on p-STAT3 and SOX2 protein levels (**Supplementary Figure 1A-C**). Inhibition of the STAT3/SOX2 signaling axis dramatically reduced the DUSP3 downregulation-promoting effects on osteosarcoma cell proliferation, migration, and invasion (**Supplementary Figure 1D-I**). Similarly, STAT3 inhibition significantly altered the stemness-promoting effect of DUSP3 knockdown in osteosarcoma cells (**Supplementary Figure 1J-K**). Thus, our results indicated that DUSP3 regulates the STAT3/SOX2 axis in osteosarcoma.

### DUSP3 binded to EGFR and inhibited its phosphorylation level

**Figure 6:**
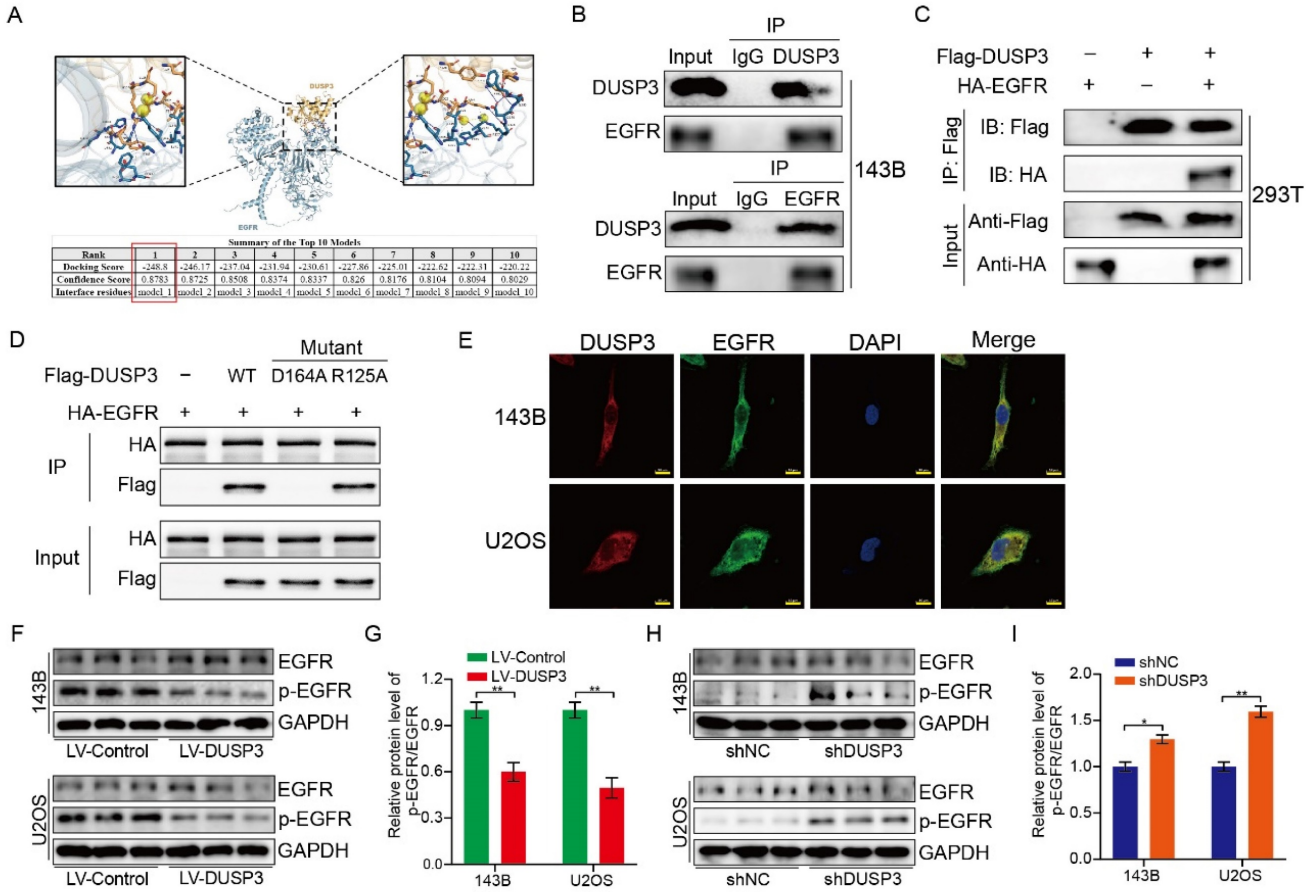
DUSP3 binds to EGFR and inhibits its phosphorylation level. (A) The molecular docking technique shows that DUSP3 binds to EGFR. The best binding model is model 1, with a binding score of -248.8 and a confidence score of 0.8783. (B) Immunoprecipitation experiments show that DUSP3 binds to EGFR endogenously in 143B cells. (C) Immunoprecipitation experiments show that DUSP3 binds exogenously to EGFR in 293T cells. (D) Mutations of R125A and D164A were transfected into osteosarcoma cells; co-immunoprecipitation experiment was performed to detect functional fragments. R125A: the arginine at position 125 was replaced by alanine; D164A: the aspartic acid at position 164 was replaced by alanine. (E) Detection of co-localization of DUSP3 and EGFR under confocal microscopy. Scale bar: 10 μm. (F-G) Overexpression of DUSP3 inhibits EGFR phosphorylation. (H-I) DUSP3 knockdown increases the phosphorylation of EGFR. Student's t-test (two groups) and one-way ANOVA (more than two groups) were employed to analyze group differences. The means ± standard deviations (SD) are used to illustrate all data. *P <0.05, **P <0.01.

To investigate the specific mechanisms, we used molecular docking techniques and found that EGFR might interact with DUSP3 and regulate osteosarcoma stemness (**Figure 6A**). To verify whether DUSP3 interacts with EGFR, we transfected DUSP3 and EGFR plasmids into 143B and 293T cells, respectively. In 143B cells, DUSP3 interacted with EGFR in an IP assay (**Figure 6B**). Similarly, when we transfected flag-tagged DUSP3 and HA-tagged EGFR into 293T cells, we found that DUSP3 interacted with EGFR (**Figure 6C**). Our results indicate that DUSP3 binds to EGFR endogenously and exogenously. By analyzing the protein structure of DUSP3, we predicted that 164-ASP and 125-ARG in DUSP3 are involved in the interaction between DUSP3 and EGFR. Therefore, we constructed related mutants. 164-ASP is essential for the interaction between EGFR and DUSP3, according to IP experiments (**Figure 6D**). Additionally, immunofluorescence co-localization experiments showed that DUSP3 interacts with EGFR (**Figure 6E**). As DUSP3 has dephosphorylase activity, we speculated that it could dephosphorylate EGFR, inhibiting the activation of key downstream molecules. According to western blot analysis, upregulation of DUSP3 significantly impaired the phosphorylation of EGFR (**Figure 6F-G**), whereas knockdown of DUSP3 promoted the phosphorylation of EGFR (**Figure 6H-I**). Thus, our results confirmed that DUSP3 interacts with EGFR and inhibits its phosphorylation.

### DUSP3 regulated the stemness of osteosarcoma cells via EGFR/STAT3/SOX2 axis

**Figure 7:**
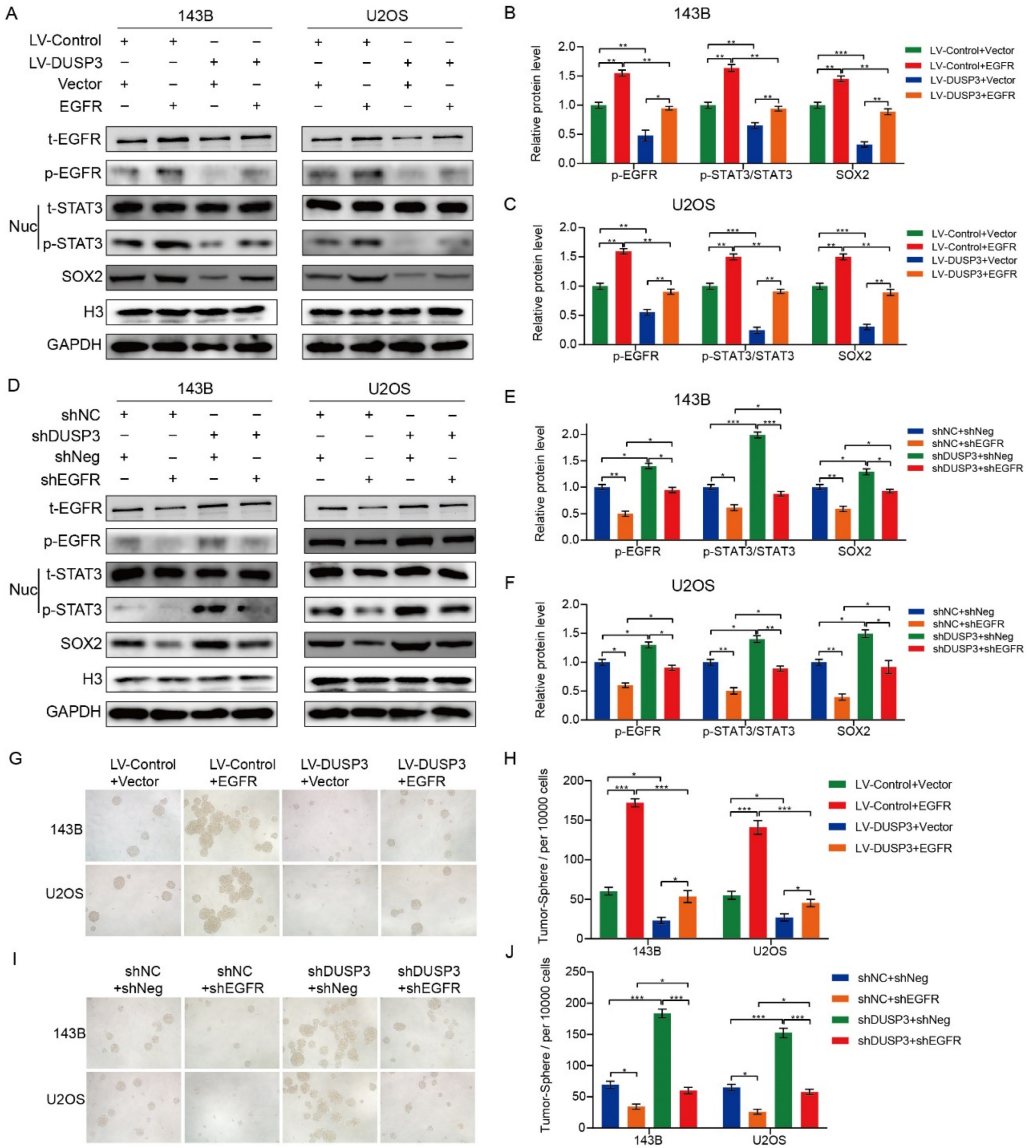
DUSP3 regulates the stemness of osteosarcoma cells via the EGFR/STAT3/SOX2 axis. (A-C) Overexpression of EGFR significantly attenuated the inhibitory effect of DUSP3 overexpression on p-STAT3 and SOX2 expression levels. (D-F) EGFR knockdown impaired the promoting effect of DUSP3 downregulation on p-STAT3 and SOX2 expression levels. (G-H) Overexpression of EGFR impaired the inhibitory effect of DUSP3 upregulation on the cancer stem cell population. (I-J) Silencing EGFR attenuated the promoting effect of DUSP3 silencing on the cancer stem cell population. For analysis of group differences, student's t-test (two groups) and one-way ANOVA (more than two groups) were used. All data are demonstrated as means ± standard deviations (SD). *P <0.05, **P <0.01, ***P <0.001.

EGFR, a growth factor receptor, has been shown to regulate the stemness of many tumors 26-28. Therefore, we speculated that DUSP3 affects the stemness of osteosarcoma cells by regulating the EGFR/STAT3/SOX2 axis. We first examined the effect of DUSP3 on the mRNA expression levels of EGFR, but did not find a significant change (**Supplementary Figure 2A-B**). As shown in **Figure 7A-C**, EGFR upregulation significantly impaired the inhibitory effect of DUSP3 overexpression on p-STAT3 and SOX2 protein expression levels. Conversely, EGFR knockdown impaired the promoting effect of DUSP3 downregulation on these proteins (**Figure 7D-F**). We compared the correlation between DUSP3, p-EGFR, and p-STAT3 in human samples and found that DUSP3 negatively regulated p-EGFR and p-STAT3 expression levels in patients (**Supplementary Figure 2C-E**). Additionally, on the population of osteosarcoma stem cells, EGFR overexpression significantly lessened the inhibitory effect of DUSP3 upregulation (**Figure 7G-H**), whereas the promoting effect of DUSP3 downregulation on osteosarcoma stem cell population was significantly inhibited upon EGFR downregulation (**Figure 7I-J**). Collectively, our results demonstrated that DUSP3 regulates the stemness of osteosarcoma cells through the EGFR/STAT3/SOX2 axis.

### DUSP3 restrains the growth and lung metastasis of osteosarcoma *in vivo*

**Figure 8:**
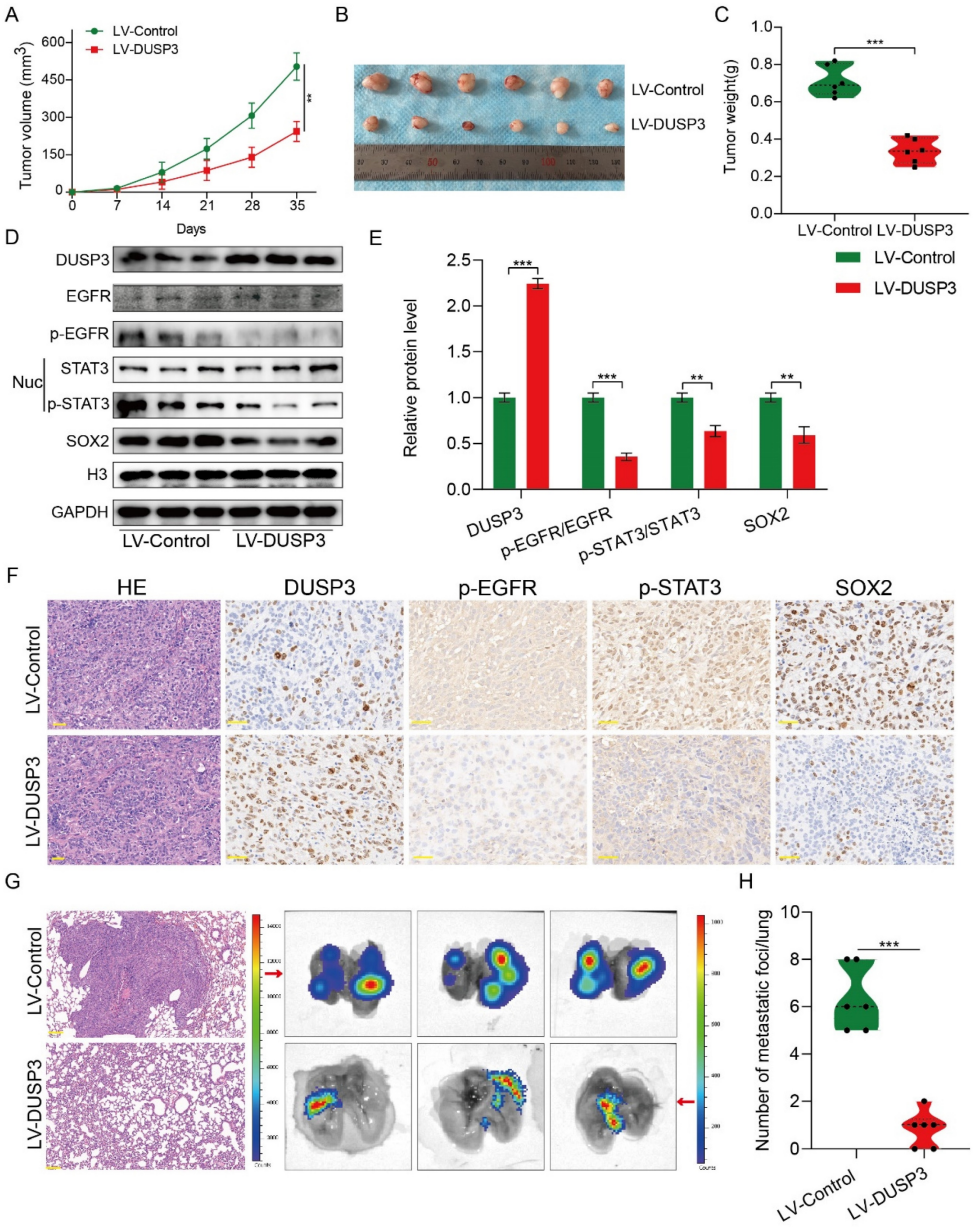
DUSP3 restrains the progression and lung metastasis of osteosarcoma in *vivo* via the EGFR/STAT3/SOX2 signaling axis. (A-C) Upregulation of DUSP3 inhibits tumor volume and weight of osteosarcoma in *vivo*. (D-E) The protein expression level of DUSP3, EGFR, p-EGFR, STAT3, p-STAT3, and SOX2 in xenograft tumors were detected by western blot. (F) The expression levels of DUSP3, p-EGFR, p-STAT3, and SOX2 in tumor samples from control and DUSP3 overexpression groups were demonstrated by IHC analysis. Scale bar: 40 μm. (G-H) HE staining and its quantitative analysis demonstrated the number of lung metastases in tumor samples from normal and DUSP3 overexpression groups. Scale bar: 200 μm. Student's t-test (two groups) and one-way ANOVA (more than two groups) were employed to analyze group differences. The means ± standard deviations (SD) are used to illustrate all data. *P <0.05, **P <0.01, ***P <0.001.

The above research prompted us to further verify the biological roles and specific mechanisms of DUSP3 using an in *vivo* model. Thus, we established xenograft models by subcutaneous injection of stably transfected 143B osteosarcoma cells. As shown in **Figure 8A-C**, tumor volume and weight in LV-DUSP3 group were smaller and lighter than in control group, indicating that overexpression of DUSP3 impairs osteosarcoma in *vivo*. Moreover, to verify EGFR/STAT3/SOX2 axis in *vivo*, we examined the expression of p-STAT3, p-EGFR, and SOX2 in the two groups of tumor tissues, confirming the significant downregulation of p-STAT3, p-EGFR, and SOX2 expression levels (**Figure 8D-E**), which was consistent with the results of IHC staining (**Figure 8F**). This indicates that the EGFR/STAT3/SOX2 axis was significantly inhibited in* vivo*. Additionally, we investigated the effect of DUSP3 overexpression on the distant metastasis of osteosarcoma in *vivo* using a mouse lung metastasis model by injecting stably transfected osteosarcoma cells into mice through the tail vein. DUSP3 overexpression group demonstrated a remarkable reduction in lung metastases, as observed by visual observation and hematoxylin-eosin staining, suggesting that the overexpression of DUSP3 significantly inhibited lung metastasis in osteosarcoma (**Figure 8G-H**). In conjunction with our earlier in *vitro* research, we can infer that DUSP3 inhibits osteosarcoma development and lung metastasis in *vivo* through regulating EGFR/STAT3/SOX2 axis.

## Discussion

Previous studies have shown that DUSP3 regulates ubiquitinated degradation of OCLN by inhibiting the tyrosine phosphorylation level of OCLN, thereby regulating metastasis in lung adenocarcinoma 29. DUSP3 affects P53 levels by binding to Y29, Y67, and Y271 of the nucleophosmin protein, regulating the genomic stability of cells after UV radiation 30. Reportedly, DUSP3 dysfunction is closely related to the regulation of renal ischemia/reperfusion 31. Despite DUSP3 research in other fields has progressed, its mechanism in osteosarcoma is still unknown.

In this study, we examined the expression levels of DUSP3 in normal and tumor tissues and discovered that DUSP3 was markedly downregulated in tumor cells and tissues. We then performed univariate and multivariate Cox regression, ROC curve, and Kaplan-Meier survival analyses. The findings demonstrated that DUSP3 was highly accurate in predicting the outcome of osteosarcoma patients, and that patients with low expression of DUSP3 had a less favorable prognosis. To test this hypothesis, we constructed osteosarcoma cell lines stably transfected with DUSP3 knockdown or overexpression. We found that DUSP3 overexpression significantly inhibited the progression of osteosarcoma cells, whereas its knockdown showed the opposite effect. Thus, these results suggest that DUSP3 exerts an inhibitory effect on osteosarcoma and may serve as an independent prognostic factor.

The JAK/STAT signaling pathway is a conserved transmembrane signal transduction pathway that plays a complex and critical role in regulating the proliferation, differentiation, apoptosis, and angiogenesis of tumor cells 32, 33. Jiang et al. revealed that RP11-468E2.5 inhibits the JAK/STAT signaling pathway by regulating the phosphorylation of STAT5 and STAT6, which ultimately suppresses growth and promotes apoptosis of colorectal cancer cells 34. In osteosarcoma, LINC01116 was found to activate the JAK/STAT signaling pathway by regulating the mir-520a-3p/IL6R axis, thereby promoting the progression and metastasis of osteosarcoma 35. Transcriptome sequencing revealed that DUSP3 expression level was closely related to the STAT3/SOX2 signaling pathway, which may be the specific mechanism by which DUSP3 restrains the malignant behaviors of osteosarcoma. Stattic, a STAT3 inhibitor, was treated with osteosarcoma cells after transfecting with shDUSP3. Furthermore, we found that treatment with Stattic significantly attenuated the promoting effects of DUSP3 knockdown on osteosarcoma cell proliferation, migration, invasion, and stemness.

EGFR is involved in maintaining the biological behavior of epithelial tissues, regulating the development of several tumors, and plays a vital role in the treatment of various tumors 36, 37. Lu et al. showed that EGFR promotes breast cancer development via STAT3-mediated transcriptional regulation 38. The EGFR-STAT3 axis is significantly upregulated in glioblastomas overexpressing circular E-cadherin, and the activation of this signaling axis significantly promotes glioblastoma malignancy 39. Hepatocellular carcinoma cells are resistant to lenvatinib by activating the EGFR-STAT3-ABCB1 axis 40.

After applying the molecular docking technique and referring to several studies, we speculated that DUSP3 affects the stemness of osteosarcoma cells by regulating EGFR. Our validation using endogenous and exogenous IP experiments showed that DUSP3 interacts with EGFR and inhibits its phosphorylation. To verify whether the EGFR/STAT3/SOX2 axis was activated, we altered the expression levels of DUSP3 and EGFR and found that p-STAT3 and SOX2 expression levels were significantly altered. Additionally, we measured the tumorsphere formation ability in both cell lines to confirm whether this signaling axis regulates DUSP3-mediated osteosarcoma cell stemness. Our results showed that DUSP3 binds to EGFR, inhibiting its phosphorylation, thereby regulating STAT3/SOX2 axis and affecting osteosarcoma cell stemness.

## Conclusion

Our research identified DUSP3 as a tumor suppressor gene in osteosarcoma. DUSP3 is downregulated in osteosarcoma and its lower expression indicates poor patient outcomes. DUSP3 impairs osteosarcoma cells proliferation, migration, invasion, and stemness through regulating the EGFR/STAT3/SOX2 axis. Targeting DUSP3 and the EGFR/STAT3/SOX2 axis may offer a new therapeutic approach for treating osteosarcoma.

## Supplementary Material

Supplementary figures.

## Figures and Tables

**Table 1:** Clinical correlation analysis of DUSP3 protein expression in osteosarcoma patients.

Characteristics	Total	DUSP3 expression		*P* value
		High	Low	
Gender				*P = 0.756*
Male	17	7	10	
Female	14	5	9	
Age				*P= 0.5915*
≤18 years	23	9	14	
>18 years	8	4	4	
Tumor Location				*P=0.7879*
Femur	13	7	6	
Tibia	6	4	2	
Humerus	5	2	3	
Others	7	3	4	
Histological types				*P=0.8064*
Osteoblastic	17	8	9	
Chondroblastic	8	3	5	
Others	6	2	4	
Clinical stage				P=0.0291*
I/II	21	13	8	
III	10	2	8	

**Table 2:** Details of the antibodies

Antibodies	Company	Catalog number	Species	Application	Dilutions
DUSP3	CST	4752	Rabbit	WB, IP	1:1000, 1:50
DUSP3	Abcam	EPR5492	Rabbit	IHC	1:500
GAPDH	Servicebio	GB15004	Rabbit	WB	1:1000
N-cadherin	CST	13116	Rabbit	WB	1:1000
E-cadherin	CST	3195	Rabbit	WB	1:1000
Vimentin	Servicebio	GB11192	Rabbit	WB	1:1000
OCT4	Proteintech	11263-1-AP	Rabbit	WB	1:1000
SOX2	CST	3579	Rabbit	WB	1:1000
SOX2	Servicebio	GB11249	Rabbit	IHC	1:1000
Nanog	ABclonal	A22115	Rabbit	WB	1:1000
p-STAT3	ABclonal	AP1468	Rabbit	WB	1:1000
p-STAT3	Servicebio	GB150001	Rabbit	IHC	1:1000
t-STAT3	Servicebio	GB11176	Rabbit	WB	1:1000
H3	Servicebio	GB11102	Rabbit	WB	1:1000
p-EGFR	Servicebio	GB114199	Rabbit	WB	1:1000
t-EGFR	ABclonal	A2069	Rabbit	WB, IP	1:1000, 1:100
