# Supplementary material for: DUSP3 restrains the progression and stemness property of osteosarcoma through regulating EGFR/STAT3/SOX2 axis
Source: Int J Biol Sci. 2025 Jan 1;21(1):160–74. doi: 10.7150/ijbs.101137 (PMC11667805; doi:10.7150/ijbs.101137)
Supplement: Supplementary file 1 — Supplementary figures. [file ijbsv21p0160s1.pdf]

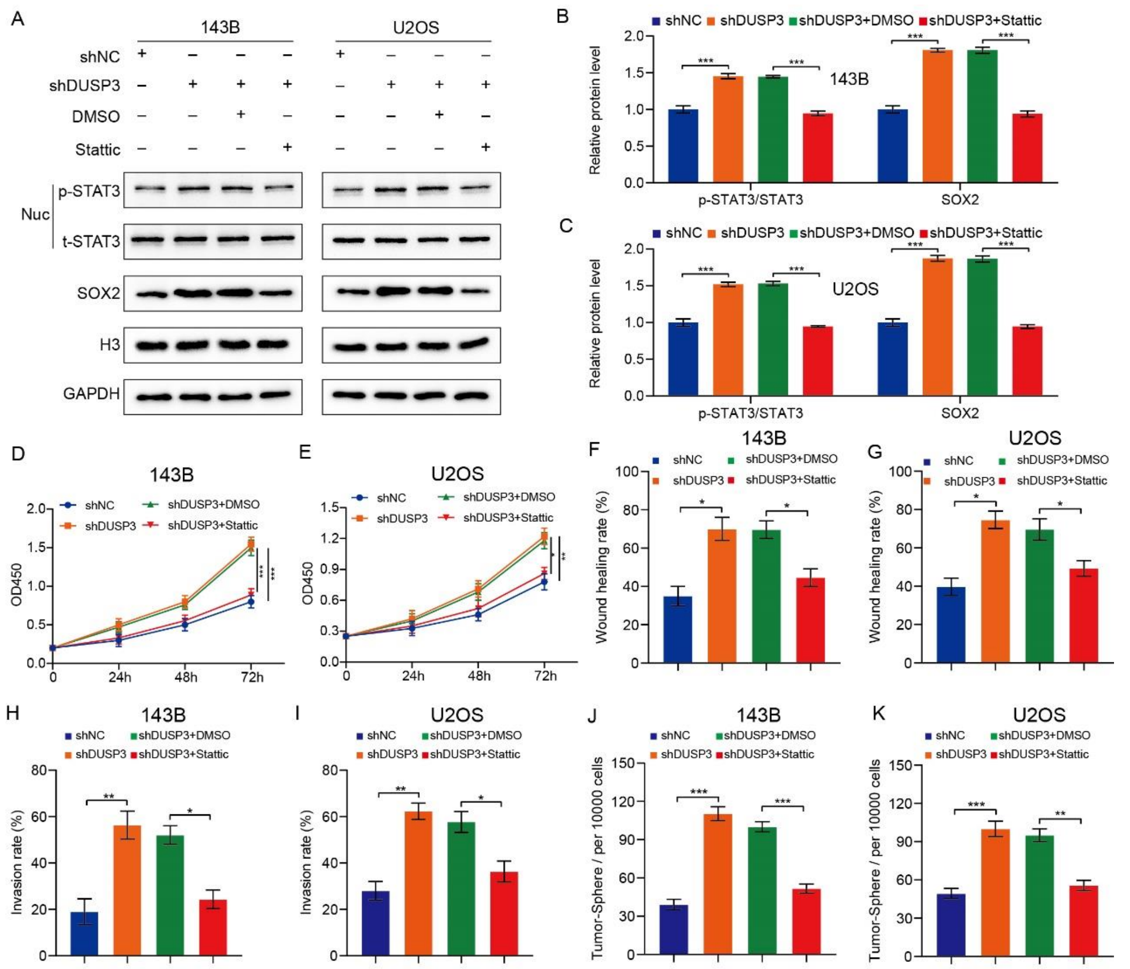

Supplementary Figure 1. DUSP3 regulates the STAT3/SOX2 axis in osteosarcoma. (A-C) Western blot analysis was applied to detect the expression levels of STAT3 and SOX2. (D-E) Stattic treated cells were assayed for cell activity by CCK-8 assay. (F-G) Stattic treated cells were applied for cell migration by wound-healing assay. (H-I) Stattic treatment significantly attenuated the promoting effect of DUSP3 knocking down on the invasion of osteosarcoma cells. (J-K) Stattic treatment altered the stemness-promoting effect of knockdown of DUSP3 on osteosarcoma cells. For analysis of group differences, student's t-test (two groups) and one-way ANOVA (more than two groups) were used. All data are demonstrated as means ± standard deviations (SD). **P* <0.05, ***P* <0.01, ****P* <0.001.

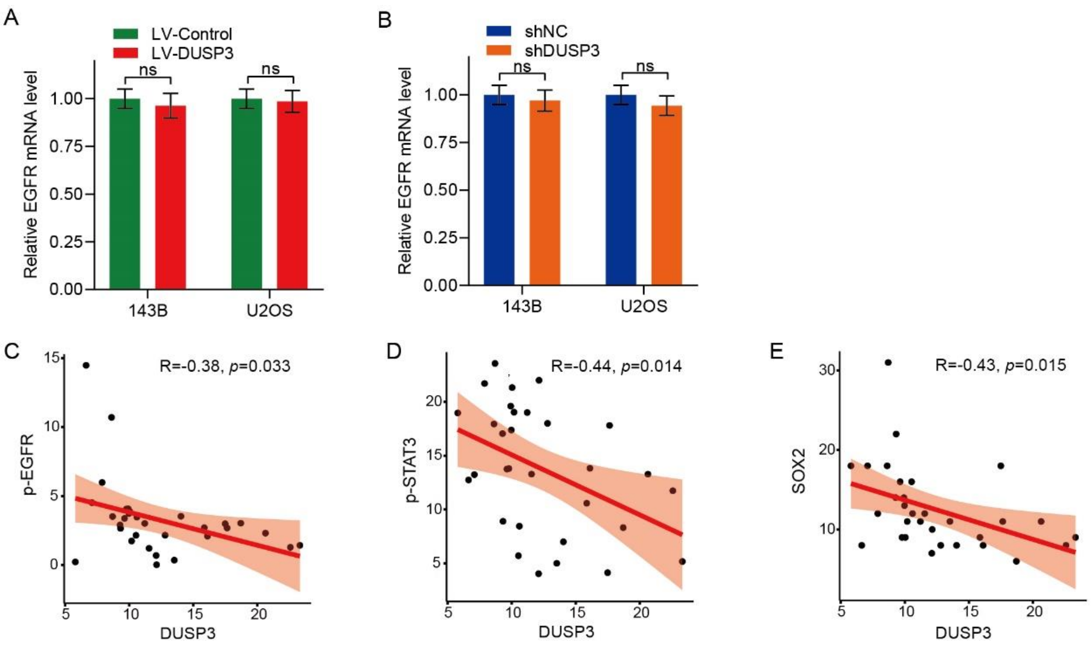

Supplementary Figure 2. DUSP3 negatively regulates the protein expression levels of p-EGFR and p-STAT3. (A-B) mRNA levels of EGFR after overexpressing or silencing of DUSP3. (C-E) The correlation between DUSP3 and p-EGFR, p-STAT3, SOX2 in human tissues were detected. Student's t-test (two groups) and one-way ANOVA (more than two groups) were employed to analyze group differences. The means ± standard deviations (SD) are used to illustrate all data.
